# Microbial interactions in facilitating antibiotic activity and resistance evolution

**DOI:** 10.1128/aem.01931-25

**Published:** 2026-01-05

**Authors:** Lujie Zhang, Shenmiao Li, Ziqi Liu, Run-run Zhang, Tian Yang, Donghong Liu, Tian Ding, Xiaonan Lu, Jinsong Feng

**Affiliations:** 1 College of Biosystems Engineering and Food Science, Zhejiang University 12377, Hangzhou, China; 2 Department of Food Science and Agricultural Chemistry, McGill University 549338, Sainte-Anne-de-Bellevue, Québec, Canada; 3 Future Food Laboratory, Zhejiang University Innovation Center of Yangtze River Delta, Zhejiang University 723177 , Jiaxing, China; 4 National-Local Joint Engineering Research Center of Intelligent Food Technology and Equipment, Zhejiang University 12377, Hangzhou, China; The Pennsylvania State University, University Park, Pennsylvania, USA

**Keywords:** microbial interactions, antibiotic activity, antibiotic resistance, ecological context

## Abstract

In polymicrobial communities, microorganisms do not exist in isolation but engage in complex and dynamic interactions. Emerging evidence indicates that these microbial interactions can profoundly influence key aspects of antibiotic action, including antibiotic activity and the emergence and dissemination of antibiotic resistance. This mini-review examines the mechanistic pathways through which intra- and inter-specific interactions facilitate both individual and community-level responses to antibiotic treatment. Such interactions can also reshape the selective pressures imposed by antibiotics, thereby altering evolutionary trajectories toward resistance. We emphasize the importance of considering the ecological context of microbial communities as essential for advancing our understanding of antibiotic resistance and for developing more effective and sustainable antibiotic strategies.

## INTRODUCTION

Conventional antibiotic susceptibility testing is performed using monoculture assays under highly controlled and homogeneous conditions (1). However, in clinical infections, the complex ecological context of pathogens often involving polymicrobial communities can lead to substantial alterations in antibiotic susceptibility profiles. A key factor underlying these changes is interspecies interaction, which significantly influences community structure and function, thereby modulating the effectiveness of antibiotic treatments (2, 3). Indeed, recent studies have shown that microbial interactions involving both pathogens and commensal species can either enhance or impair antibiotic activity, ultimately altering the susceptibility of focal species or the entire community. Consequently, the response of a bacterial strain to antibiotic treatment within a community may significantly diverge from its behavior in isolation. In some cases, species classified as antibiotic-susceptible in monoculture may persist or even thrive within a community, while antibiotic-resistant species may exhibit inhibited growth (4). These findings suggest that antibiotic susceptibility and resistance evolution are emergent properties of microbial communities, shaped not only by the intrinsic traits of individual species but also their interactions with neighboring microorganisms.

Two key community-level phenomena arising from these interactions are cross-sensitization and cross-protection (5). Cross-sensitization is an emergent communal behavior in which a bacterial species that is tolerant to an antibiotic in isolation becomes more susceptible when embedded in a microbial community. In contrast, cross-protection refers to a phenomenon in which a species that is susceptible to isolation can nonetheless survive and proliferate in the presence of an antibiotic due to protective effects conferred by coexisting species. This outcome does not arise from genetic adaptation but rather from one of two mechanisms: either the protective community extrinsically modifies the microenvironment (e.g., by reducing the effective antibiotic concentration experienced by the susceptible strain) or the susceptible strain exhibits reversible, non-mutational physiological or metabolic adjustments in response to the altered conditions. Notably, these two definitions are not mutually exclusive. They may occur simultaneously within a community, resulting in complex and dynamic patterns of antibiotic response. Such intra- and inter-species interactions can influence not only the immediate effectiveness of antibiotic treatments but also the selective pressures that drive the evolution of antibiotic resistance. By modulating the relative fitness of susceptible and resistant strains, microbial interactions can shape growth dynamics, alter fitness costs associated with resistance, and affect long-term evolutionary trajectories (6). In this review, we discuss how microbial interactions within multi-species communities influence antibiotic activity and contribute to the emergence and evolution of antibiotic resistance genes (ARGs).

## MICROBIAL INTERACTIONS IN MULTI-SPECIES COMMUNITIES

Microbial species rarely exist in isolation; instead, they typically coexist in close proximity with others (7). This coexistence drives complex interplay among different microbial species that influence community assembly, behavioral and evolutionary outcomes (8). Understanding the collective survival of microbial communities under antibiotic pressure requires insight into how these intricate microbial interactions shape such traits.

### Types of microbial interactions

**Fig 1:**
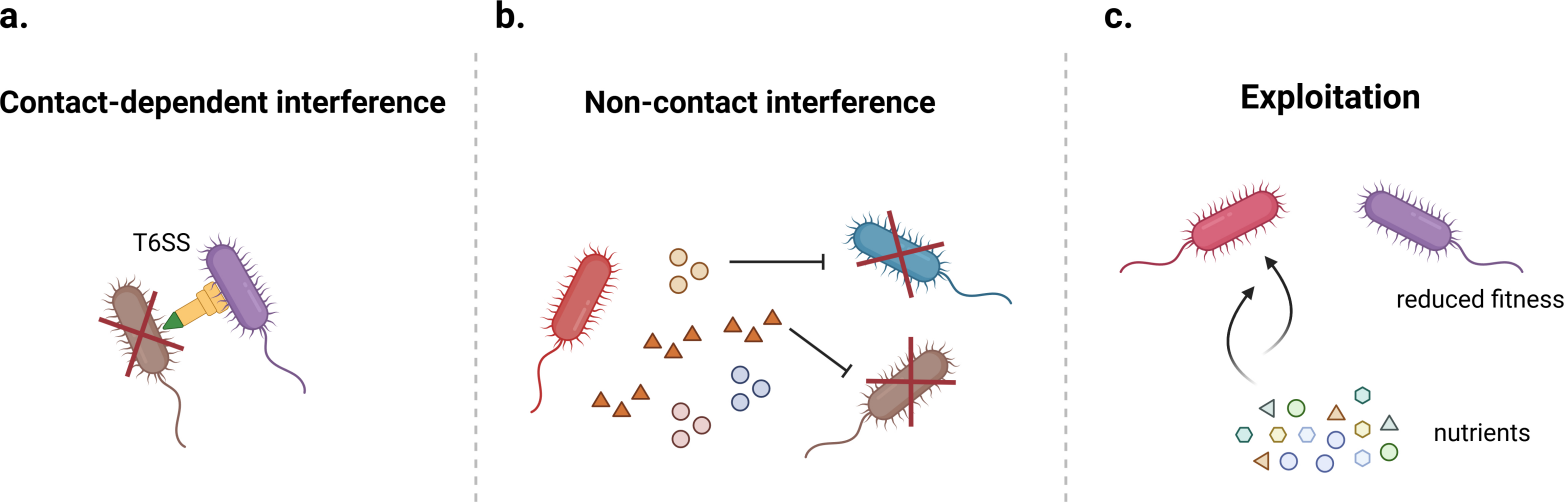
Direct and indirect interspecies interactions of microbial competition. (**a**) Contact-dependent interference includes mechanisms such as type VI secretion system-mediated killing. (**b**) Non-contact interactions include inhibition mediated by the secretion of antimicrobial metabolites. (**c**) Exploitation describes the competition for limited resources among microorganisms. Illustration created using BioRender.

**Fig 2:**
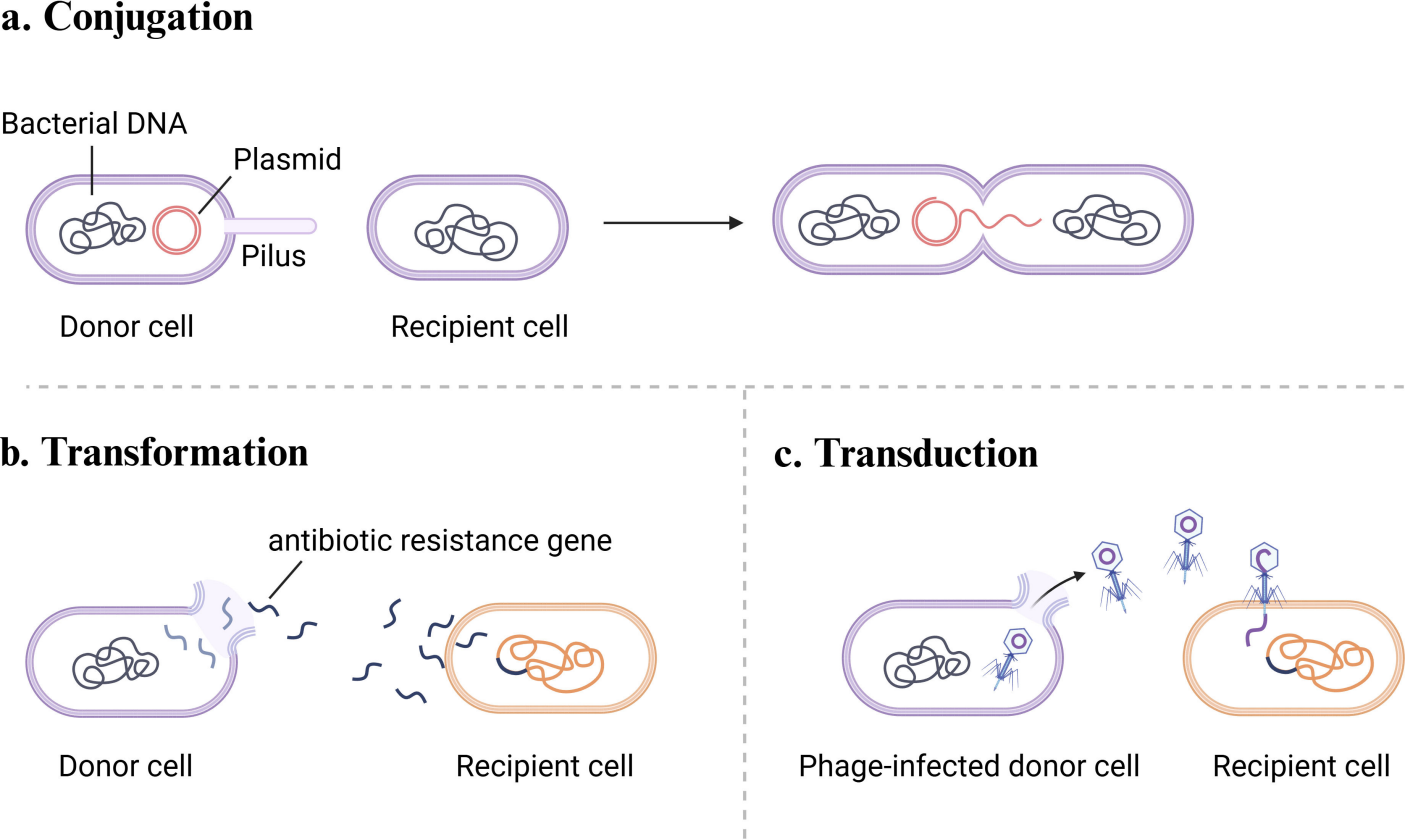
Primary mechanisms of horizontal gene transfer (HGT) that enables bacteria to acquire resistance genes from other bacteria, facilitating rapid spread of antibiotic resistance. (**a**) Conjugation involves direct physical contact between donor and recipient cells, mediated by a conjugative pilus that facilitates the transfer of genetic material. (**b**) Transformation involves the uptake of free extracellular DNA from the environment by recipient cells, which can then incorporate the exogenous genetic material into their genomes. Illustration created using BioRender. (**c**) Transduction refers to the phage-mediated transfer of bacterial genetic material between cells.

Bacterial communities are intricately shaped by a diverse array of interactions, which can be systematically categorized as either direct or indirect, depending on whether physical contact is required or the interaction occurs via environmental mediators such as metabolites, signaling molecules, or genetic material (9). Direct interactions involve physical cell-to-cell contact or close spatial proximity. An example is contact-dependent interference, such as type VI secretion system (T6SS), which enables bacteria to engage in competitive interactions by delivering toxic effectors directly into rival cells (10) (Fig. 1a). Another form of direct interaction is conjugation, one of the three primary mechanisms of horizontal gene transfer (HGT) in prokaryotes, which requires direct contact between donor and recipient cells (Fig. 2a). This process is mediated by a conjugative pilus that mediates the transfer of genetic material. Conjugation is most prevalent in densely packed environment, such as biofilms, where close cell proximity enhances the efficiency of gene exchange (11–13). Indirect interactions occur through the release and detection of chemical signals or metabolites in the environment, without requiring physical contact between bacterial cells. A common example is quorum sensing (QS), a cell-density-dependent communication system in which bacteria secrete signaling molecules that accumulate in the surrounding environment. Once a threshold concentration is reached, these molecules trigger coordinated behaviors within the bacterial community (14).

**Fig 3:**
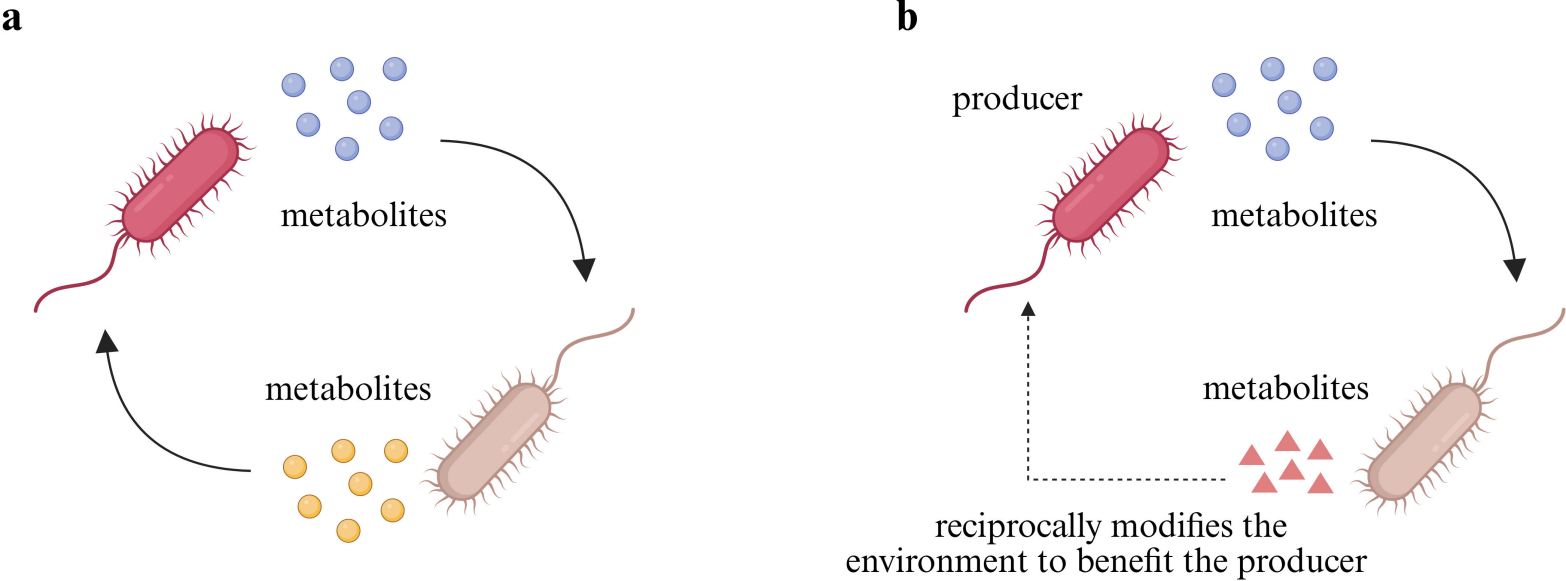
Metabolic cross-feeding mediates indirect inter-species interaction. (**a**) Direct cross-feeding involves the mutual exchange of metabolites between microbial species. (**b**) Indirect cross-feeding occurs when one microbe uses a metabolite produced by another and subsequently modifies the environment in a way that benefits the original producer. Illustration created using BioRender.

Another key form of indirect interaction is metabolic cross-feeding, also known as syntrophy. In this process, two microbial species either exchange metabolites for mutual benefit or one species uses a metabolite produced by another while simultaneously modifying the environment in a way that benefits the producer (15) (Fig. 3). Cross-feeding has been found across various ecological settings. For example, in the human gut microbiome, Pérez et al. (16) revealed that cross-feeding of amino acids among a variety of species, particularly Firmicutes such as *Clostridium*, *Actinomyces*, *Peptostreptococci,* and *Propionibacterium*, plays a crucial role in maintaining nitrogen balance. However, the outcomes of cross-feeding are highly context dependent. For instance, Huus et al. (17) demonstrated that in a carbohydrate-rich but protein-deficient diet, *Bacteroidales* spp. and *Enterobacteriaceae* spp. form a cross-feeding network involving sugars and iron metabolites. This interaction promotes bacterial overgrowth, potentially perturbing the equilibrium of gut microbiota and increasing the risk of infection. Given this context-dependent nature, it is crucial to systematically understand how cross-feeding influences population dynamics and responses at the community level to environmental changes, such as antibiotic treatment. Beyond these interactions, non-contact interference competition is another type of indirect interaction. In this case, microorganisms secrete antimicrobial compounds or other inhibitory metabolites to suppress or inactivate competing species (18) (Fig. 1b). Alternatively, exploitation competition arises when microorganisms compete for limited resources. Competitive advantage is gained through superior efficiency in resource acquisition, which reduces availability of others and negatively impacts their fitness (Fig. 1c). Finally, transformation, a key mechanism of HGT, is also a significant indirect interaction mechanism. In this process, bacteria or archaea assimilate exogenous DNA from their surroundings and incorporate it into their genomes, potentially acquiring new traits such as antibiotic resistance (19, 20) (Fig. 2b). Similarly, transduction is a phage-mediated process where bacterial DNA is transferred between cells through phage particles (Fig. 2c).

This classification of bacterial interactions into direct and indirect types is crucial for understanding the complex behaviors within bacterial communities. However, in natural settings, these interactions rarely occur in isolation; instead, they often operate concurrently and are integrated into complex networks. For example, quorum sensing and metabolic cooperation can coexist within a biofilm, contributing to the formation of a dynamic and multifaceted microbial ecosystem (21–23). Investigating these interactions can provide valuable insights into how bacterial communities function, adapt, and respond to environmental changes.

## MICROBIAL INTERACTIONS AND THEIR IMPACT ON ANTIBIOTIC ACTIVITY: CROSS-SENSITIZATION AND CROSS-PROTECTION

Cross-sensitization refers to increased antibiotic susceptibility of a microbe when it resides within a microbial community, compared to its susceptibility in isolation (5). For instance, competitive interactions between antibiotic-resistant and -susceptible bacteria specifically in the form of exploitation competition for shared resources can result in spatial segregation. Resistant bacteria that can degrade antibiotics often cluster together, thereby isolating themselves from susceptible bacteria. This spatial separation prevents susceptible bacteria from accessing the protective benefits from the detoxification activities of the resistant bacteria, thereby exposing them to higher antibiotic concentrations and increasing vulnerability to antibiotic stress (24). In addition to competition, metabolic cross-feeding has also been documented to affect the antibiotic effectiveness (3, 25). This metabolic interdependence can make microbial communities more susceptible to perturbations compared to their constituent species in monoculture. In such cases, the overall antibiotic tolerance of the bacterial community is often determined by the least tolerant member, known as the “weakest link” principle (26, 27). Targeting these metabolic interactions could represent a promising strategy to improve antibiotic activity, provided that its efficacy and safety are further validated in clinical studies. For example, Adamowicz et al. (3) found that an obligate cross-feeding consortium including *Salmonella enterica*, *Escherichia coli,* and *Methylobacterium extorquens* displayed greater susceptibility to tetracycline and ampicillin compared to the individual species grown in monoculture. Even in non-obligate cross-feeding systems, similar dynamics can occur. Anaerobic mucin-degrading bacteria that metabolize mucin into short-chain fatty acids (e.g., propionate and acetate) support the growth of *Pseudomonas aeruginosa*. Ampicillin can indirectly inhibit the growth of *P. aeruginosa* by targeting the mucin-degrading bacteria, thereby reducing the availability of essential fatty acids. This metabolic interdependency increases the susceptibility of *P. aeruginosa* to antibiotics (3). Table 1 summarizes the variations in antibiotic susceptibility and the underlying mechanisms of the selected bacterial species under monoculture and community conditions.

**TABLE 1:** Examples of variations in antibiotic susceptibility between monoculture and community conditions for selected bacterial species and their underlying mechanisms^*a*^

Species	Antibiotic	Monoculture MIC	Community MIC	Effect type	Mechanism
*E. coli*	Ampicillin	2 µg/mL	1 µg/mL	Cross-sensitization	Dependent on metabolic by-products from *S. enterica* and *M. extorquens*
	Tetracycline	2 µg/mL	4 µg/mL	Cross-protection	Delayed growth in cooperative community allows for tetracycline breakdown
*S. enterica*	Ampicillin	1 µg/mL	1 µg/mL	No change	No significant change in MIC
	Tetracycline	50 µg/mL	4 µg/mL	Cross-sensitization	Dependent on metabolic by-products from *E. coli* and *M. extorquens*
*M. extorquens*	Ampicillin	100 µg/mL	1 µg/mL	Cross-sensitization	Dependent on metabolic by-products from *E. coli* and *S. enterica*
	Tetracycline	5 µg/mL	No growth	Cross-sensitization	Dependent on metabolic by-products from *E. coli* and *S. enterica*
*P. aeruginosa*	Ampicillin	>25 µg/mL	5 µg/mL	Cross-sensitization	Dependent on metabolic by-products from mucin-degrading anaerobes

^*a*^ Data in this table are from Adamowicz et al. (3).

### Antibiotic inactivation as a mechanism of cross-protection

**Fig 4:**
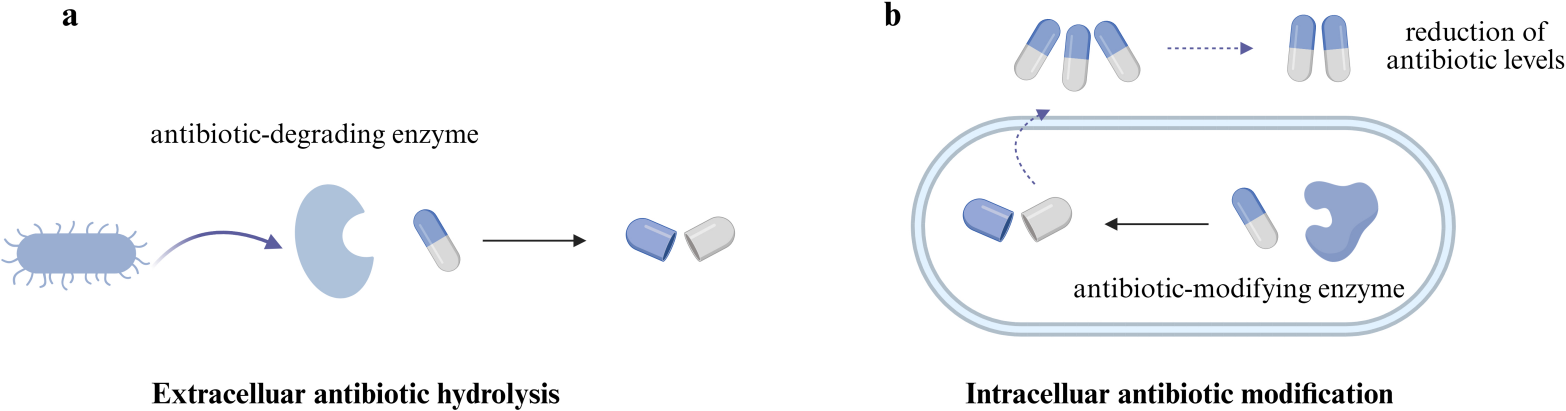
Antibiotic inactivation-mediated cross-protection. (**a**) Secretion of antibiotic-degrading enzymes can generate cross-protection effect to susceptible neighboring bacteria. (**b**) Intracellular antibiotic modification involves the uptake and antibiotic neutralization of antibiotics to indirectly reduce antibiotic levels in the surrounding environment. Illustration created using BioRender.

Compared to cross-sensitization, cross-protection is a more frequently observed scenario in community contexts (5). This protection often stems from a facilitative microbial interaction known as community-mediated antibiotic inactivation, in which the physiological response of one species alters the environment to support the survival of rest susceptible species. The process operates through two distinct routes: extracellular neutralization and intracellular denaturation of antibiotics. Extracellular antibiotic inactivation is typically mediated by the secretion of antibiotic-degrading enzymes (Fig. 4a). For example, β-lactamase-secreting *E. coli* strains can significantly enhance the survival of ampicillin-susceptible strains of *S*. Typhimurium without any detectable genetic transfer (28). Importantly, this protective effect does not require direct cell-to-cell contact, indicating that β-lactamase diffusion into the surrounding environment is sufficient to reduce antibiotic concentrations and confer protection to the neighboring cells.

In addition to extracellular antibiotic hydrolysis, intracellular antibiotic modification also plays a role in context-dependent antibiotic inactivation (Fig. 4b). For instance, bacteria expressing chloramphenicol acetyltransferase can efficiently inactivate chloramphenicol via intracellular enzymatic modification, reducing the concentration of the active antibiotic available in the surrounding environment. The resulting reduction of chloramphenicol concentration can support the survival of chloramphenicol-susceptible *Streptococcus* pneumococci, allowing genetically susceptible strains to survive and potentially outcompete resistant species (29). Another study found that the production of aminoglycoside-modifying enzymes by *P. aeruginosa* can lead to the inactivation of aminoglycoside antibiotics such as gentamicin and tobramycin coculture environments, potentially offering protection to other species against the antibiotic’s lethal effects (30).

### Exoproduct-mediated protection

Under the selective pressure of antibiotics, bacteria have evolved a variety of strategies to survive, including cross-protection mechanisms mediated by the exoproducts of neighboring species. These exoproducts modify the metabolic processes of recipient bacteria, enhancing their survival under antibiotic challenge (31, 32). This exoproduct-mediated protection represents another significant form of indirect microbial interaction. For example, *P. aeruginosa* secretes 2-*n*-heptyl-4-hydroxyquinoline *N*-oxide (HQNO), a compound that inhibits the electron transport chain in *Staphylococcus aureus*. HQNO forces *S. aureus* to shift its metabolism from respiration to fermentation, leading to slower growth and reduced susceptibility to antibiotics targeting cell wall synthesis and protein production, such as vancomycin (31). Similarly, HQNO-mediated disruption of the electron transport chain diminishes the proton motive force in *S. aureus*, a necessary prerequisite for aminoglycoside uptake, thereby conferring protection against antibiotics such as tobramycin (32). This form of bacterial altruism, where one species endures a fitness cost to produce and share protective exoproducts, highlights the complex cooperative dynamics that can emerge within microbial communities.

### Biofilm-associated cross-protection

Biofilms are structured communities of microbial cells that adhere to surfaces or to each other, typically enclosed in a self-generated extracellular matrix (33). These communities exhibit complex ecological dynamics that significantly influence the development, maturation, stability, and biomass of both mono- and multi-species biofilms. Direct interactions, such as cell-to-cell contact, can facilitate the initial adhesion and aggregation of bacterial cells (34). Indirect interactions, particularly the secretion of signaling molecules via quorum sensing, are vital for coordinating bacterial population-level behaviors. These signaling molecules enable bacteria to sense their population density and initiate actions, such as surface adhesion, microcolony formation, and expansive growth in both lateral and vertical dimensions (35, 36). For instance, in a study by Wang et al. (37), high levels of the quorum sensing molecule autoinducer-2 (AI-2) prompted *Pseudomonas fluorescens* and *S. aureus* to generate substantial quantities of exopolysaccharide, facilitating the coaggregation of these two bacterial species and development of intricate, dual-species biofilms with elaborate spatial structures. Consequently, the biofilm cells demonstrated reduced susceptibility to carvacrol.

**Fig 5:**
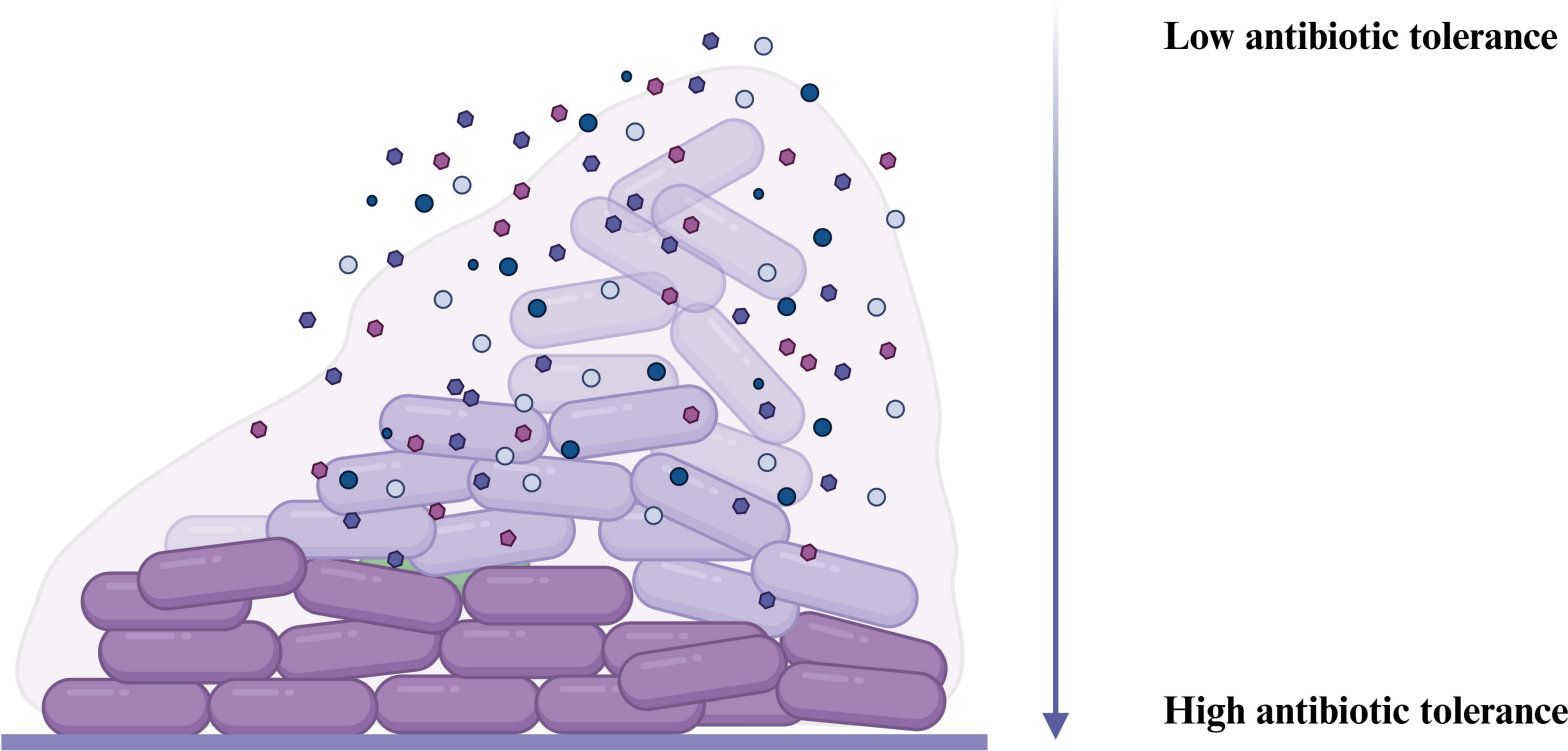
Heterogeneous antibiotic diffusion in biofilms. The biofilm microenvironment is characterized by a descending gradient of oxygen and nutrient availability from the exterior toward its interior. This spatial gradient leads to reduced bacterial metabolic activity and increased antibiotic tolerance. Illustration created using BioRender.

For certain antibiotics, the biofilm matrix, which is predominantly composed of water, does not present a generic barrier to the diffusion of solutes that are of a size comparable to antibiotics (38, 39). However, the penetration of an antibiotic into a biofilm may be impeded if microorganisms within the biofilm form aggregates that interfere with its diffusion (40, 41). In such cases, the antibiotic is deactivated in the surface layers at a rate that exceeds its rate of diffusion. Consequently, cells located deeper within the biofilm are subjected to significantly lower antibiotic concentrations, increasing their likelihood of survival (42, 43). Likewise, cells at the center of biofilms frequently encounter restricted oxygen and nutrient availability, leading to lower metabolic activity and decreased susceptibility to antibiotics (44) (Fig. 5).

Biofilm ecosystems comprise a complex structure with distinct ecological niches. The colonization patterns within these niches are strongly shaped by both intra- and inter-specific interactions. Ecologically, such interactions ranging from interspecific cooperation (e.g., public goods sharing, nutrient cross-feeding, detoxification, and gene transfer) to interspecific competition (e.g., exploitative and interference competition) profoundly affect the fitness of individual microbial cells and the functionality of the community. These dynamics give rise to emergent functions and behaviors that are absent in single-species biofilms. For example, the presence of *Haemophilus influenzae* markedly increases both biofilm formation and antibiotic tolerance in *Moraxella catarrhalis*, facilitating its persistence within polymicrobial biofilm communities (45). Similarly, the extracellular matrix produced by *Candida albicans* during biofilm formation has been demonstrated to provide a protective niche for *S. aureus*, shielding it from antibiotic-induced killing *in vivo* (46). Other cooperative interactions, such as extracellular electron transfer, may also contribute to these emergent properties. For example, *Streptococcus gordonii* produces hydrogen peroxide, which induces *Aggregatibacter actinomycetemcomitans* to reorganize its spatial positioning to minimize oxidative damage. This adaptation also increases oxygen availability, thereby shifting the metabolism of *A. actinomycetemcomitans* from fermentation to respiration, which is a process known as cross-respiration. This metabolic shift improves energy yield and enhances the overall fitness of *A. actinomycetemcomitans*, strengthening its persistence under environmental stress (47). Based on these findings from dual-species biofilms, there has been a growing interest in studying more complex dynamics of multispecies biofilms. For example, Burmølle and colleagues discovered that a four-species biofilm exhibited greater metabolic activity in the presence of tetracycline compared to single-species biofilms, suggesting that interspecies interactions enhance overall biofilm fitness and survival during antibiotic challenge (48). Similarly, in multispecies biofilms comprising *Streptococcus anginosus*, *S. aureus*, and *P. aeruginosa*, the effectiveness of antibiotics is notably diminished compared to that observed in monospecies counterparts. This attenuation is primarily attributed to protective interspecies interplay. For example, the killing of *S. anginosus* by cell wall-targeting antibiotics was significantly reduced in the multispecies context (49). This observation underscores the biofilm-specific nature of antibiotic resistance. Nonetheless, the precise mechanisms underlying these interactions are still not clear. Further investigation into the intricate dynamics within the multispecies biofilm ecosystem is needed.

### Microbial interactions affect the development of antibiotic resistance

Antibiotic resistance has emerged as one of the most critical public health challenges of the 21st century, posing a severe global threat (50, 51). In response, antibiotic resistance surveillance programs and research initiatives have been established worldwide. While effective phenotypic surveillance remains essential, advanced genetic and genomic approaches including long-read metagenomics, artificial intelligence (AI)-driven surveillance systems, and phylodynamic analysis are playing an increasingly important role (52). A deeper understanding of microbial community dynamics can further strengthen these efforts by revealing the complex interactions among antibiotic-resistant pathogens, thereby improving the effectiveness of surveillance and mitigation strategies. Antibiotic resistance is an inheritable trait that arises from genetic mutations or acquisition of resistance genes, conferring a stable and transmissible tolerance to antibiotics (53). Both direct and indirect microbial interactions can significantly influence the development and spread of antibiotic resistance via multiple mechanisms. For instance, since genetic and environmental factors facilitate the effects of mutations, microbial interactions can influence the mutation supply by affecting population size and mutation rates (54, 55). These interactions also have the potential to reshape the adaptive landscape, thereby influencing the fitness effects of beneficial mutations (56–59). Overall, microbial interactions play a dual role in shaping the development of antibiotic resistance. On one hand, they can facilitate the emergence and spread of antibiotic-resistant strains under certain ecological conditions. On the other hand, they may constrain the proliferation of resistant microorganisms within complex communities.

### Microbial interactions facilitate antibiotic resistance development

A key mechanism through which microbial interactions facilitate the dissemination of antibiotic resistance is HGT. HGT enables bacteria to obtain resistance genes from other bacteria, thereby accelerating the dissemination of resistance both within and across species. The three primary modes of HGT in prokaryotes are conjugation, transformation, and transduction (60) (Fig. 2). Among these, plasmid-mediated conjugation is the most predominant driver of antibiotic resistance gene transfer (61). One mechanism by which bacteria accelerate gene transfer is through the predation of neighboring cells to acquire exogenous genetic elements. Cooper et al. (62) demonstrated that when *Acinetobacter baumannii* and *E. coli* are co-cultured in the presence of kanamycin, *Acinetobacter* acts as a potent predator. Using its T6SS, it lyses *E. coli* cells and captures kanamycin resistance genes. This adaptive form of HGT via cross-species competence not only supports the survival of pathogenic bacteria under antibiotic stress but also enables their proliferation in new ecological niches, thereby intensifying the challenge of antibiotic resistance. In addition to competition-driven mechanisms, inter- and intra-specific cooperative plays a critical role in facilitating HGT. Fu et al. (63) applied co-occurrence pattern analyses to investigate the associative dynamics between *Vibrio* species and related taxa in aquacultural environments. Their findings revealed frequent resistance gene exchanges among these cooperative species, which substantially contributed to rapid emergence of resistant populations. These insights highlight the importance of considering both competitive and cooperative interactions in the dissemination of antibiotic resistance genes. Such dynamics are relevant not only in environmental settings such as aquaculture but also in the human gut, a vast genetic reservoir where HGT occurs at a 25-fold higher frequency among human-associated bacteria than between microbes from disparate environments (64). This elevated rate of gene transfer, independent of phylogenetic relatedness, accelerates bacterial adaptation to antibiotics and environmental stressors (65, 66).

Some studies have also revealed a strong connection between bacterial communication and antibiotic resistance (67, 68). For example, *S*. Typhimurium shows increased antibiotic tolerance within polymicrobial settings due to indole, a signaling molecule produced by *E. coli* that activates stress-response pathways in *S*. Typhimurium (69). Likely, *Pseudomonas putida* can detect indole secreted by *E. coli* within mixed microbial communities, which triggers the activation of an efflux pump regulated by indole, enabling *P. putida* to grow in the presence of ampicillin (70). Altruistic communal interactions extending beyond interspecies dynamics are also common within intraspecific populations. For example, under antibiotic stress, highly resistant *E. coli* mutants can synthesize and secrete indole, despite the associated fitness cost. In turn, indole activates drug efflux pumps and oxidative stress defense mechanisms, thereby enhancing the survival of less resistant members of the population (71). These findings underscore the importance of bacterial signaling molecules in facilitating antibiotic resistance through both inter- and intra-species interactions.

Studies using conditioned media as a proxy for microbial interactions further illustrate their impact on antibiotic resistance evolution. In uropathogenic isolates such as *E. coli*, *Klebsiella pneumoniae*, and *Enterococcus faecium*, the trajectory of resistance development varies depending on the microbial context. Resistance evolves most slowly when bacteria are grown in their own conditioned medium, suggesting that competition among conspecifics with high niche overlap exerts weaker selective pressure for antibiotic resistance (72, 73). In contrast, resistance evolves more rapidly when bacteria are cultured in the conditioned medium of phylogenetically distant species (74). These observations suggest that niche-specific effects, such as resource partitioning and ecological differentiation, mediated by indirect microbial interactions, can shape the dynamics of antibiotic resistance evolution.

### Microbial interactions constrain antibiotic resistance development

Microbial interactions can effectively constrain the spread of antibiotic resistance by narrowing the ecological niche of resistant bacteria, reducing their fitness and limiting their colonization potential within microbial communities. Although antibiotic-resistant strains may be genetically stable, they often carry a fitness cost, typically manifested as reduced growth rates (75). This disadvantage is further amplified in community settings, where interactions such as resource competition further suppress the growth and persistence of resistant strains (76). Consequently, the metabolic burden associated with resistance can diminish the selective advantage of these resistant strains in the presence of antibiotics, thereby impeding the dissemination of antibiotic resistance genes.

Cross-feeding interactions also play a significant role in shaping the evolutionary dynamics of antibiotic resistance. In such interactions, the survival of one species may depend not only on its inherent resistance phenotype but also on the resistance profiles of its metabolic partners. The “weakest link” effect can limit the development and diversification of antibiotic resistance mechanisms. For example, studies have shown that obligate cross-feeding interactions significantly slow the rate of adaptation to antibiotics in *S. enterica* and *E. coli* compared to monoculture conditions (77). Moreover, the community context can alter evolutionary trajectories, leading to the emergence of distinct resistance mechanisms in monoculture vs co-culture environments. Obligate metabolic interdependencies not only decelerate resistance development but also alter the type and extent of resistance mutations that ultimately arise.

## CONCLUSIONS AND PERSPECTIVES

Recent studies have increasingly emphasized the importance of considering both the growth environment and the composition of co-colonizing microbiota in shaping the susceptibility of pathogens to antibiotic treatment. The response of pathogens is influenced not only by their genetic makeup but also by their interactions with surrounding microorganisms (5). As a result, antibiotic susceptibility measured in monoculture may not accurately predict outcomes within the complex and multispecies communities. This discrepancy is an emergent property of microbial ecosystems and manifests in two contrasting phenomena, namely, cross-sensitization and cross-protection. Multiple mechanisms could drive these behaviors. Cross-sensitization can result from spatial segregation, which prevents susceptible cells from accessing detoxifying enzymes produced by neighboring organisms or from disruptions in obligate cross-feeding relationships. In contrast, cross-protection may occur through antibiotic degradation via extracellular hydrolysis or intracellular modification, metabolic shifts induced by secreted exoproducts from co-habiting microbes, or the induction of biofilm-mediated tolerance. Collectively, these mechanisms underscore the intricate interplay between microbial interactions and antibiotic responses in polymicrobial environments.

Beyond influencing immediate antibiotic activity, microbial interactions also shape the long-term evolutionary trajectories of antibiotic resistance (74). Mechanisms such as HGT, intra- and inter-specific signaling, and niche-specific ecological pressures contribute to the spread of antibiotic resistance. Conversely, evolutionary constraints such as the fitness costs of resistance, restricted ecological niches, and metabolic dependencies in cross-feeding networks can slow or redirect the adaptation process. These dynamics reveal the need for a system-level understanding of antibiotic resistance, where interplay at multiple scales produces complex and sometimes counterintuitive outcomes.

Understanding the key mechanisms underlying changes in drug susceptibility and the development of resistance requires elucidating microbial interactions within multispecies populations. Experimental approaches such as coculture and microcosm studies enable direct observation of these interactions under controlled conditions (78, 79). Systems biology techniques, including metagenomics, metatranscriptomics, metaproteomics, metabolomics, and stable-isotope probing (SIP) combined with omics, offer integrative insights into genetic potential, gene expression, protein function, and metabolic activity (80). Computational approaches, such as network analysis and metabolic modeling, further predict and explain these interactions (81–83). By combining these complementary strategies, researchers can unravel the complex interplay among microbes in multispecies populations, providing critical ecological context for resistance development. Despite these advances, important limitations remain. Existing studies often examine a narrow range of antibiotics and microbial communities, limiting broader applicability. Future research should incorporate more diverse antibiotics and microbial populations to strengthen our understanding. In addition, emergent community-level phenomena may create discrepancies between *in vitro* observations and *in vivo* outcomes, emphasizing the need for further validation.
